# Flexural Strength Analysis of Different Complete Denture Resin-Based Materials Obtained by Conventional and Digital Manufacturing

**DOI:** 10.3390/ma16196559

**Published:** 2023-10-05

**Authors:** Alessio Casucci, Giulia Verniani, Anne Lucrèce Barbieri, Nicolò Maria Ricci, Edoardo Ferrari Cagidiaco, Marco Ferrari

**Affiliations:** Department of Prosthodontics, University of Siena, 53100 Siena, Italy; alessio.casucci@gmail.com (A.C.); giuliaverniani96@gmail.com (G.V.); annelucre.barbieri@student.unisi.it (A.L.B.); dott.n.m.ricci@gmail.com (N.M.R.); edoardo.ferrari.cagidiaco@gmail.com (E.F.C.)

**Keywords:** 3D printing, flexural strength, complete dentures

## Abstract

PMMA (Polymethylmethacrylate) is the material of choice to fabricate denture bases. Recently, with the introduction of CAD-CAM and 3D printers in dentistry, new materials have been proposed for complete denture manufacturing. Aim: This study compared the flexural strength of different resins fabricated using different technologies (conventional, CAD-CAM-milled, and 3D-printed) and polymerization techniques. Methods: A total of 11 different resins were tested: six PMMA conventional (Acrypol R, Acrypol LL, Acrypol HI, Acrypol Fast, Acryself and Acryslef P), two milled obtained from UDMA PMMA disks (Ivotion disk and Aadva disk, control groups), two 3D-printed PMMA resins (NextDent Denture 3D+, and SprintRayEU Denture Base), and one 3D-printed composite resin (GC Temp Print). Flexural strength was measured using a universal testing machine. One-way ANOVA and Bonferroni post hoc tests were performed; the *p*-value was set at 0.05 to consider statistically significant differences among the groups. Spearman test was used to evaluate the correlation between polymerization technique and the flexural strength of 3D-printed resins. Results: CAD-CAM-milled specimens showed the highest flexural strength (107.87 MPa for UDMA) followed by 3D-printed composite resins (102.96 MPa). Furthermore, 3D-printed resins polymerized for 40 min with the BB cure unit showed no statistically significant differences with conventional resin groups. Moreover, in all the 3D-printed specimens, a high correlation between polymerization technique and flexural strength was found. Conclusions: In terms of flexural strength, the polymerization technique is a determinant for both acrylic and composite resins. Temp Print can be a potential alternative to fabricating removable dentures and showed promising results when used in combination with pink color resin powder.

## 1. Introduction

Removable complete dentures represent, for edentulous patients, the least invasive and most cost-effective prosthodontic rehabilitation [1]. Acrylic resins have been the material of choice for denture bases since they were introduced in dentistry by Dr. Walter Wright and the Vernon Brothers in Philadelphia in 1937. To this day, PMMA (polymethylmethacrylate) remains the most used acrylic resin for denture base fabrication [2]. The PMMA used in the dental field is conventionally obtained by mixing a liquid and a powder. The powder is composed of repolymerized polymethylmethacrylate particles as well as a peroxide initiator. The liquid component is made of a cross-linking agent, an inhibitor, and a monomer of methyl methacrylate (MMA). In the transparent powder, pigments and other substances, such as acrylic synthetic fibers and nylon, are added to imitate oral tissues [3].

PMMA gained popularity due to its good physicochemical properties as well as its low cost and acceptable aesthetics [4,5]. Nevertheless, there have been increasing concerns about some characteristics of this material, such as the frequent fractures of dentures [6], polymerization shrinkage, and cytotoxicity [3,7]. For instance, the addition of nanoparticles and nanotubes was tested to improve the material’s mechanical properties [8,9,10,11]. To overcome polymerization shrinkage found in heat-cured and cold-cured resins, injection molding was introduced [12,13]. Chemical changes were tested to be stabilizers of PMMA, but newer innovative methods have yet to be investigated [14].

The introduction of computer-aided design/computer-aided manufacturing technology (CAD-CAM) and 3D printers in the dental field added new possibilities to improve the materials and workflows used for denture fabrication. Some of the advantages of CAD-CAM fabrication are a decreased denture weight and lower resin volume, two qualities that can increase the patient’s comfort [15]. Moreover, the issue of polymerization shrinkage was eliminated thanks to the use of pre-polymerized discs, leading to a better adaptation fit and higher mechanical performance. In fact, in the milling technique, an already polymerized block is milled to the final dimensions [16,17].

In terms of 3D-printing technologies, the most commonly used in dentistry are stereolithography apparatus (SLA) and digital light processing (DLP), which are two different photopolymerization devices. Once the CAD model is converted into an STL file, it is sliced into different layers and then built one layer at a time. To complete a layer, the SLA technique cures it line by line using a laser beam, whereas DLP cures layer by layer using a projector. This makes the DLP technique faster and less prone to errors caused by repeated printing. Post-processing, defined by cleaning the object and post-curing, is different according to each technology and recommendation of the manufacturer [18,19,20,21].

In the last few years, the mechanical properties of both acrylic and composite 3D-printed resins have been investigated in the dental field. Printed composite resins are mostly used for temporary crowns and bridges, and promising results in terms of flexural strength, fracture load, and hardness have been found [22,23]. Moreover, composite resins such as urethane dimethacrylate (UDMA) showed good dimensional stability and a lack of residual monomers, reducing the risk of contact allergies [24,25]. For these reasons, UDMA could be considered a suitable alternative for denture base fabrication. Therefore, PMMA-milled resin-based materials were included in the present study as control groups for the evaluation of different resin-based materials’ mechanical behavior. 

Concerning the mechanical properties, flexural strength is the most frequent test applied to dental materials, along with impact strength and hardness. Flexural strength is the combination of compressive, tensile, and shear stress and is defined as the maximum stress that a material experiences at its yielding point. This test is fundamental in the evaluation of denture base materials as it gives an indication of the material’s resistance to fracture and a prediction of its behavior under static loads. High values of flexural strength will reduce the risk of denture base fractures. Conforming to the ISO-20795-1:2013 [26] recommendations, the three-point bending test is the most commonly used to assess the flexural strength of polymers [27,28]. Many studies have been conducted to compare the flexural strengths of denture base materials fabricated analogically and through CAD-CAM technologies using the three-point bending test [22,29,30,31,32,33,34,35]. 

The mechanical performance of resin composites is closely related to their formulation [36,37]. The molecular backbone characteristics of the co-monomers involved will determine the hydrophilicity, mobility, and kinetic parameters. When acrylic resin strengths are compared, those with a lower degree of conversion exhibit inferior mechanical properties [38]. The higher flexural strength values of CAD-CAM specimens may be attributed to a higher degree of conversion [15].

Nevertheless, despite the numerous investigations on the advantages of digital workflow, few studies exist comparing conventional, CAD-CAM subtractive, and additive manufacturing methods at the same time [34,35]. In this study, the aim was to compare the flexural strengths of denture base resins fabricated conventionally, CAD-CAM-milled and 3D printed with different polymerization techniques. The null hypothesis was that there is no statistically significant difference in flexural strength between the different tested materials.

## 2. Materials and Methods

**Figure 1:**
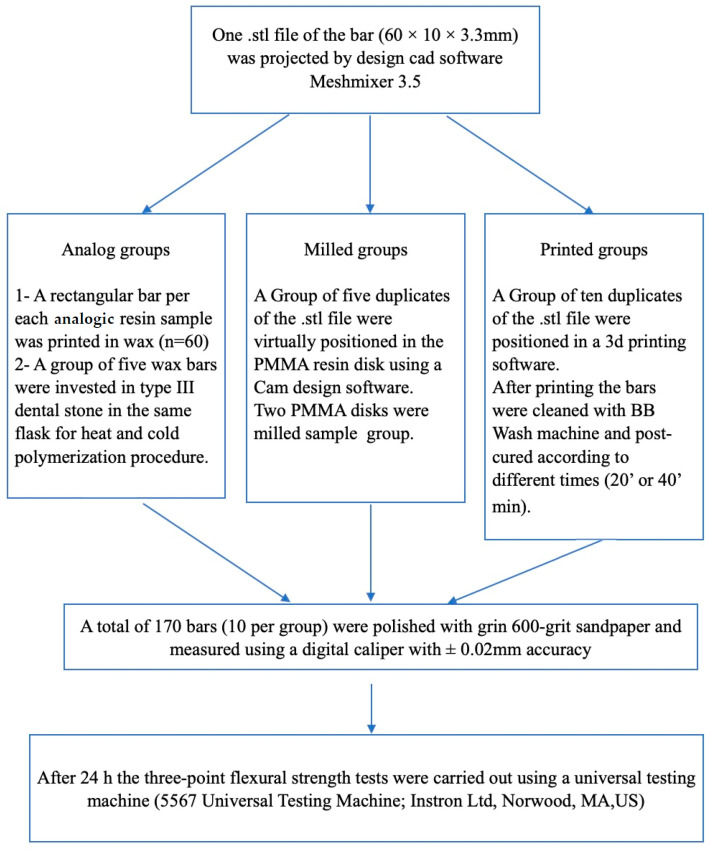
Schematic diagrams on sample fabrication method and procedures reported for the different sample groups.

In this study, 170 rectangular specimens of resin-based material having dimensions of 64 × 10 × 3.3 mm were fabricated according to the ISO-20795-1:2013 standard [26]. Twelve different resins were used: Six analog acrylic resins, two PMMA-milled resins, two 3D-printed acrylic resins, and one composite 3D-printed resin, as described in Table 1. They were divided according to the type of fabrication (analog, CAD-CAM-milled, or 3D-printed) and their polymerization method, as reported in Figure 1. Each group was composed of 10 specimens.

### 2.1. Analog Process

The analog group was composed of different resins: four resins were heat-cured (Acrypol R, Acrypol LL, Acrypol HI, and Acrypol Fast) and two were cold-cured (Acryself P and Acryself), as described in Table 2. All resins were produced by the same manufacturer, Ruthinium-Dental Manufacturing S.p.A., Rovigo, Italy.

They were fabricated according to the manufacturer’s instructions with a 3:1 powder-to-liquid ratio, except Acryself P, which had a 2:1 powder-to-liquid ratio. To fabricate these resins, a wax die was 3D printed with rectangular shapes of accurate dimensions. Once mixed, the resin was then poured inside the die, which was itself placed in a flask. It should be noted that two different flasks were used for the fabrication of Acrypol LL.

Four resins were heat-cured (Acrypol R, Acrypol LL, Acrypol HI, and Acrypol Fast), and two were cold-cured (Acryself P and Acryself), as described in Table 2.

The heat-cured resins were prepared by applying 3 tons/6000 lbs pressure to the flask. While fore the cold curing resins, the polymerization was carried out in a pot at a pressure of 2 ATM (standard atmosphere) for 10 min at a temperature of 45 °C.

### 2.2. CAD-CAM and Milling Process

For the milled group, rectangular specimens of accurate dimensions were designed using the CAD software MESHMIXER 3.5. It was then saved as a Standard Tessellation Language (STL) and sent to the milling machine. Pre-polymerized PMMA discs were fixed on a sectioning machine and milled using a diamond saw.

### 2.3. 3D Printing and Curing Process

Three types of 3D-printed resins were used: NextDent Denture 3D+ (NextDent B.V., Soesterberg, The Netherlands), GC Temp Print (GC Corporation, Tokyo, Japan), and SprintRayEU Denture Base (SprintRay Inc., Los Angeles, CA, USA).

The same STL file as the milled groups was used for the 3D-printed group. It was sent to the DLP printer Asiga MAX UV (wavelength = 385, pixel resolution = 62) and printed at a 0° build orientation, as suggested by the findings of Dai et al., 2023 [39]. After the printing process, the specimens were cleaned with Liquidtech BT for 20 min using the BB Wash machine (Meccatronicore S.R.L., Pergine Valsugana, TN, Italy). The resins then received different types of post-curing procedures (as described in Table 3).

SprintRayEU Denture Base Group (SprintRay Inc.) was further divided into two subgroups of *n* = 10 according to the polymerization technique used. One was polymerized for 20 min using the LED curing unit “LaboLight DUO” (GC Corporation, Tokyo, Japan), and the other was polymerized for 40 min using a BB cure machine (Model MTC-BB-CURE-COMPACT, Meccatronicore S.R.L., Pergine Valsugana, TN, Italy). The same procedure was carried out with the NextDent Denture 3D+ resin (NextDent B.V.).

The GC Temp Print specimens were divided into 3 subgroups depending on the polymerization procedure used: one group was polymerized for 20 min with the LaboLight DUO curing unit, the second group was polymerized for 20 min with the BB cure unit, and the third group was polymerized for 40 min with the BB cure unit.

Since GC Temp Print resin is white, another experimental group was made, mixing 3 mL of Formlabs color pigment (color MAGENTA) and 300 mg of GC Temp Print resin to reach acceptable esthetics. This resin was also divided into 2 subgroups of *n* = 10, with one group being polymerized for 20 min with the BB cure unit and one for 40 min with the BB cure unit.

### 2.4. Fabrication Accuracy

Once the curing procedure was completed, a slow-speed rotary instrument was used to remove excesses and the specimen’s support structures. All specimens were polished with a 600-grit sandpaper and measured using a digital caliper with ±0.02 mm accuracy. Before performing the test, they were stored in distilled water for 24 h.

### 2.5. Flexural Strength Analysis

**Figure 2:**
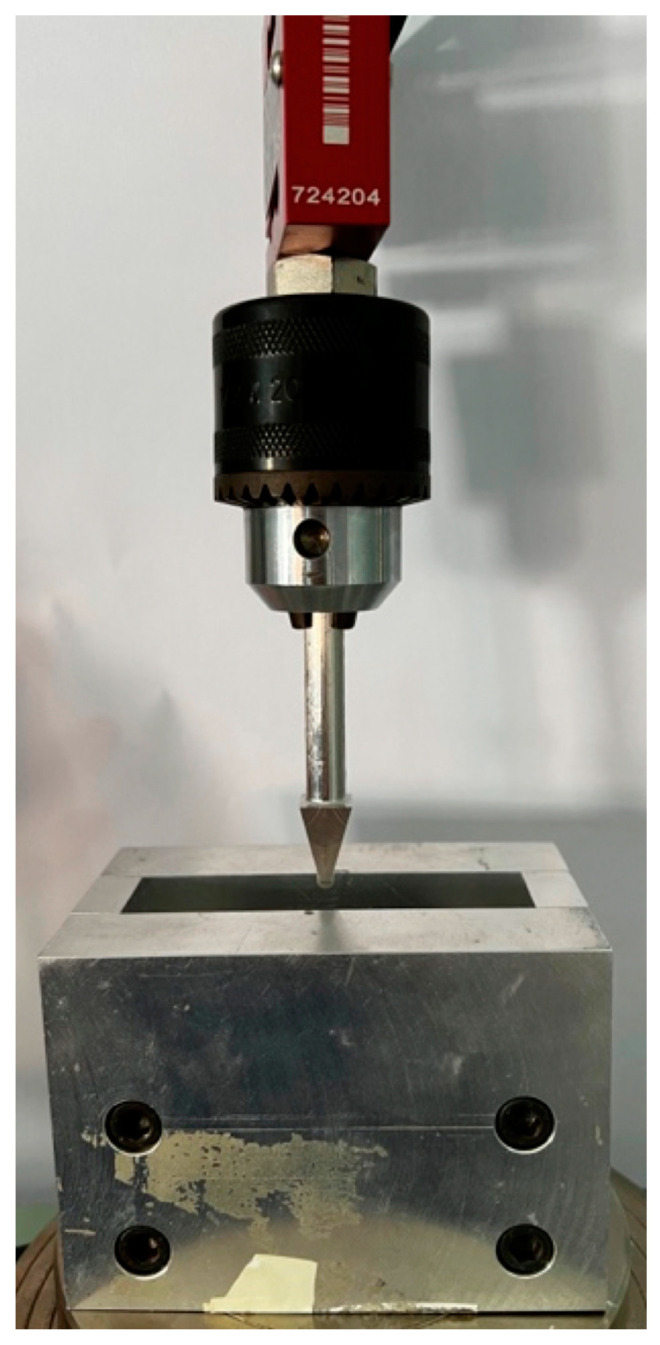
Universal machine for three-point bending test.

The three-point flexural strength tests were carried out using a universal testing machine (5567 Universal Testing Machine; Instron Ltd., Nordwood, MA, USA), placing each specimen on circular support beams with a 50 mm span as reported in Figure 2. The loading force was applied to the center of each specimen at a crosshead speed of 5 mm/min. The fracture load was recorded, and the flexural strength was then calculated in megapascals (MPa) using the following formula:

FS = (3 P L)/(2 b d^2^)

FS: flexural strength, P: maximum load, L: span length (50 mm), b: width and d: thickness.

All measurements and tests were carried out by the same operator.

### 2.6. Statistical Analysis

Statistical analysis was carried out using Statistical Package for the Social Sciences (SPSS) software, version 26 (IBM SPSS statistics, v. 26, Inc., Chicago, IL, USA). Means and standard deviations were calculated for each group. The normality was tested using a Kolmogorov–Smirnov, which confirmed a normal distribution of data. One-way ANOVA and Bonferroni post hoc tests were then performed. All *p*-values < 0.05 were considered statistically different. A Spearman correlation test was also used to measure the correlation between the polymerization technique and the flexural strength of 3D-printed resins.

## 3. Results

**Figure 3:**
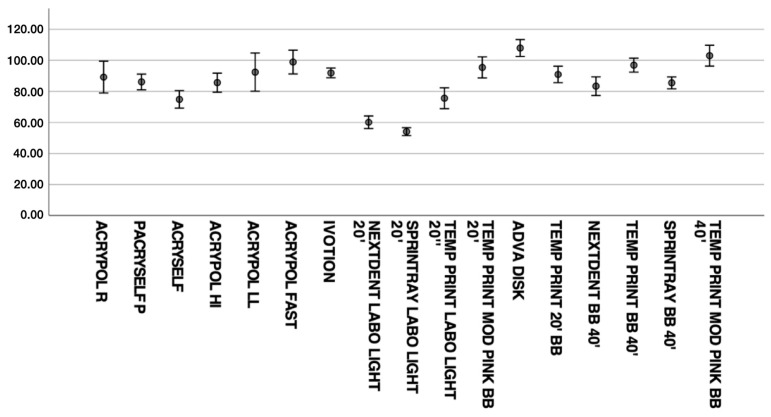
Flexural strength means (MPa) for each resin type.

Table 4 shows the mean flexural strengths and standard deviations (SD) for each group of resin, as well as the ANOVA and *p*-value. In Figure 3, all the flexural strength means are reported. Of all the tested groups, the AADVA disc had the highest mean flexural strength (107.87 MPa), and Sprintray Denture Base 3D-printed specimens polymerized for 20 min with the Labolight curing unit had the lowest (54.07 MPa).

For the analog group, the heat-cured Acrypol Fast had the highest mean (98.86 MPa), whereas the lowest was found for the Acryself group (74.83 MPa).

The 3D-printed group with the highest flexural strength was PINK Temp Print polymerized for 40 min with the BB cure unit (102.96 MPa).

### 3.1. Analog Group

Acrypol R (89.15 MPa) showed no statistically significant differences with the other groups except with the Nextdent and Spintray resins polymerized for 20 min with the Labolight unit (*p* < 0.001). Nextdent and Sprintray resins polymerized for 20 min with the Labolight unit showed no statistically significant difference between them (*p* = 1.000) but had a significantly lower mean than all the other groups (*p* < 0.05). Acryself (74.83 MPa) had the lowest flexural strength mean within the analog group; it was significantly lower than Temp Print resin polymerized for 20 min with the BB cure unit (*p* < 0.013), Temp Print resin polymerized for 40 min with the BB cure unit (*p* < 0.001), PINK Temp Print resin polymerized for 20 min with the BB cure unit (*p* < 0.001), and PINK Temp Print polymerized for 40 min with the BB cure unit (*p* < 0.001).

Acryself P (86.07 MPa) and Acrypol HI (85.58 MPa) were significantly lower than PINK Temp Print polymerized for 40 min with BB-cure (*p* = 0.006, *p* = 0.004, respectively).

Acrypol LL (92.39 MPa) was significantly higher than Temp Print polymerized for 20 min with the Labolight unit (*p* = 0.006).

Acrypol Fast had the highest mean (98.86 MPa) within the analog group. It was significantly higher than the Temp Print resin polymerized for 20 min with the Labolight unit (*p* < 0.001) and the Nextdent resin polymerized for 40 min with the BB cure unit (*p* = 0.020).

There were no statistically significant differences between the analog groups themselves except Acrypol Fast and Acrypol LL, which were significantly higher than Acryself (*p* < 0.001 and *p* = 0.003, respectively).

### 3.2. Milled Group

The AADVA disc’s flexural strength was significantly higher than all the other groups (*p* < 0.02) except the Temp Print group polymerized for 40 min with the BB cure unit (*p* = 0.839), the PINK Temp Print polymerized for 20 min with the BB cure unit (*p* = 0.275), and the PINK Temp Print polymerized for 40 min with the BB cure unit (*p* = 1.000). It also showed no statistically significant differences with Acrypol Fast (*p* = 1.000). Nevertheless, it was significantly higher than the Ivotion disc (*p* = 0.013).

The Ivotion discs presented a significantly higher flexural strength than the Nextdent and Sprintray groups polymerized for 20 min using the Labolight unit (*p* = 0.000) as well as the Temp print group polymerized for 20 min using the Labolight unit (*p* = 0.010). Compared to the analog group, it showed statistically significant differences only with Acryself (*p* = 0.005).

### 3.3. 3D-Printed Group Comparison

In the 3D-printed group, the lowest flexural strengths found were for Sprintray (54.07 MPa) and Nextdent (60.11 MPa) resins polymerized for 20 min with the Labolight unit. They showed no statistically significant difference between them (*p* = 1.000); nevertheless, they did show statistically significant differences with all the other 3D-printed resins (*p* < 0.04).

Nextdent and Sprintray groups, which were polymerized for 40 min with the BB-cure unit, were significantly lower than the PINK Temp Print polymerized for 40 min with the BB cure unit (*p* < 0.003).

Within the Temp Print resins, the one polymerized for 20 min with the Labolight unit had the lowest mean (75.58 MPa). It was significantly lower than the Temp Print resin polymerized for 20 min with the BB cure unit (*p* = 0.025). It was also significantly lower than the Temp Print polymerized for 40 min with the BB cure unit (*p* < 0.001) and the PINK Temp Print groups polymerized with the BB cure unit for 20 min (*p* < 0.001) and 40 min (*p* < 0.001).

Finally, the PINK Temp Print group polymerized for 40 min with the BB cure unit had the highest flexural strength (102.96 MPa) in the 3D-printed group. It was significantly higher than the Nextdent (*p* < 0.001) and SprintRay (*p* = 0.003) groups, which were polymerized for 40 min with the BB cure.

Spearman tests showed a high association between flexural strength and polymerization technique. The correlation coefficient was 0.811 for PINK Temp Print, 0.867 for Temp Print, 0.867 for Sprintray, and 0.867 for Nextdent.

## 4. Discussion

This study was conducted to compare the flexural strengths of acrylic and composite resins for denture base fabrication according to their fabrication technique (conventional, CAD-CAM-milled, and 3D-printed) and polymerization process. The results of this research revealed statistically significant differences among the resins tested. Therefore, the null hypothesis was rejected.

Concerning the results obtained in the present study, it can be speculated that the content and the degree of chain conversion during the polymerization may influence the flexural strength of the different resin-based materials evaluated.

As previously reported for PMMA resins, the polymerization process can be initiated by benzoyl peroxide, which can be activated by thermal energy (heat curing resins) or by the use of tertiary amines (cold curing resins) [40,41].

In the present study, it was confirmed that the mechanical properties of self-curing resins were lower (Acryself P and Acryself) than those made with heat-activated resins (Acrypol R, Acrypol LL, Acrypol HI, and Acrypol Fast) because of excess residual monomer.

Regarding the high flexural strength of GC TempPrint, the enhanced strength can be attributed to the fact that the UDMA material has a lower molar volume and molecular weight than alternative resins. This could enhance the preliminary methacrylate functionality of the un-polymerized material. It is highly likely that this increased functionality increases the crosslink density within the matrix of the polymer. When this occurs, polymeric resins exhibit enhanced mechanical properties, one of which is flexural strength [42].

The influence of BisEMA monomers with low viscosity and high MW on the mechanical behavior of resin composites has not been extensively investigated. However, the total replacement of BisGMA by BisEMA in composites with TEGDMA resulted in higher conversion, but no improvement was observed in flexural and diametral tensile strengths [43].

According to ISO-20795-1:2013 [26], the minimal flexural strength required for denture bases is 65 MPa. Of all the resins tested, only the Sprintray (54.07 MPa) and Nextdent (60.11 MPa) groups polymerized for 20 min with the Labolight unit did not meet such standards. Another study also found values under 65 MPa for the Nextdent resins using a different printer and printing orientation [30].

The highest values for flexural strength were found for the CAD-CAM-milled groups, which supports previous findings [33,35].

Flexural strength is affected by the degree of polymerization achieved. When acrylic resin strengths are compared, those with a lower degree of conversion exhibit inferior mechanical properties. The higher flexural strength values of CAD-CAM specimens may be attributed to a higher degree of conversion [44].

Thus, CAD-CAM-milled dentures can be considered a valid substitute for conventionally fabricated dentures.

Three-dimensionally printed resins polymerized with the BB cure unit, either for 20 or 40 min, always showed higher flexural strength compared to the resins polymerized with the Labolight unit; moreover, the Spearman test showed a high correlation between flexural strength and the polymerization technique used. This confirms that polymerization does play a role in mechanical properties [30].

Flexural strengths for Nextdent and Sprintray polymerized for 40 min with the BB cure unit showed no statistically significant differences with the analog groups except Acryself. Such a finding implies that 3D-printed fabrication can lead to similar results as those found for analog procedures while using a material with lower cost and less dependence on manual expertise. Three-dimensionally printed resins also implicate shorter chair time and working time, as a denture could be printed directly after scanning the edentulous arches or the analog impression. Additionally, the lower cost would also allow broader access to dental cures and reduce the economic issue of denture fractures, as it would be easier and cheaper to print a new denture compared with starting a full conventional process again.

Such an outcome is in accordance with al-Qarni et al. (2022) results, who conducted a similar experiment and found a mean flexural strength of (93.4 ± 10.8 MPa) for heat-cured analog resins and (56.4 ± 4.7 MPa) for NextDent specimens, which were printed with DLP technology and cured for 10 min at 60 degrees with an LC-D print box machine [31].

This study also confirmed the data previously obtained by Di Fiore et al. (2021) in a study conducted with a similar protocol. A flexural strength of 80.79 (±7.64 MPa) for heat-cured analog resins was found and of 110.23 (±5.03 MPa) for the CAD-CAM-milled PMMA block (Ruthinium Disc; Dental Manufacturing Spa). The 3D-printed Nextdent specimens showed a very similar flexural strength (87.34 ± 6.39 MPa) even though they were printed using an SLA printer and polymerized for 20 min with a light box (Moonlight; VertySystem) [34].

Temp Print resins showed higher flexural strengths than the other 3D-printed resins, which can be explained by the fact that it is a different material. It also showed statistically significant differences with the analog group. The use of a 3D-printed composite for a denture base could be a possible alternative, given that it also presents good dimensional stability. Temp Print showed good results of dimensional stability over time when used for full arch restorations [45], but more studies should be carried out when used for complete denture fabrication. It was shown that the addition of a pigment in order to have an acceptable aesthetic for the fabrication of dentures did not interfere with mechanical properties but improved the flexural strength. Since the material GC Temprint PINK is an experimental material never tested before the present study, it is mandatory to test in vitro and in vivo conditions in order to obtain a comprehensive evaluation before clinical use.

A limitation of its use for denture bases can be the lack of evidence on the bonding abilities of 3D-printed composite resins to liners, which could mean a new denture should be printed with the added modifications each time relining is needed. Nevertheless, studies were conducted to find the best surface treatments for both PMMA and UDMA to increase their bond to soft liners [46]. More studies should be carried out on the use of pink pigment on 3D-printed composites for dentures since the good flexural strength values found in this study.

In order to validate the materials for clinical use, further tests should be carried out, such as impact strength tests, surface hardness tests, and color stability evaluations, but mostly dimensional stability tests. Long-term studies or the use of thermocycling to imitate resins’ aging are needed to better understand the changes in mechanical properties over time.

Of course, in order to standardize the in vitro procedure, the specimens were prepared according to ISO-20795-1:2013, but further studies are indicated in order to evaluate the mechanical properties in the oral environment.

## 5. Conclusions

Within the limits of the present study, it can be concluded that:

- Temp Print specimens reported no statistically significant differences with both control groups, Ivotion and AADVA discs, proving that it can be a potential alternative to fabricating removable dentures.
- The experimental 3D-printed Temp Print composite showed promising results with the highest flexural strength within the combination of pink color resin.
- It was confirmed that flexural strength and polymerization methods are correlated.

## Figures and Tables

**Table 1:** Materials tested in the study.

Name	Manufacturer	Material Content	Batch n°
ACRYPOL R	Ruthinium-Dental Manufacturing S.p.A., Rovigo, Italy	Heat curing, acrylic resin (PMMA) Liquid: methyl methacrylate, ethylene dimethacrylate (EDMA) Powder: benzoyl peroxide, methyl methacrylate.	Powder: J1584 Liquid: J1571
ACRYSEL P	Ruthinium-Dental Manufacturing S.p.A., Rovigo, Italy	Polymerizable cold-curing resin (PMMA) Liquid: methyl methacrylate, ethylene dimethacrylate, N-N-dimethylparatoluidine. Powder: benzoyl peroxide, methyl methacrylate.	Powder: LOT J0086 Liquid: LOT I0727
ACRYSELF	Ruthinium-Dental Manufacturing S.p.A., Rovigo, Italy	Polymerizable cold-curing resin (PMMA) Liquid: methyl methacrylate, ethylene dimethacrylate, N-N-dimethylparatoluidine Powder: benzoyl peroxide, methyl methacrylate.	Powder: LOT J2163 Liquid: LOT I0727
ACRYPOL HI	Ruthinium-Dental Manufacturing S.p.A., Rovigo, Italy	Heat curing acrylic resin with a high molecular weight (PMMA) Liquid: methyl methacrylate, ethylene dimethacrylate. Powder: benzoyl peroxide, methyl methacrylate.	Powder: LOT H1172 Liquid: LOT J0890
ACRYPOL LL	Ruthinium-Dental Manufacturing S.p.A., Rovigo, Italy	Heat curing acrylic resin with a high molecular weight (PMMA) Liquid: methyl methacrylate, ethylene dimethacrylate. Powder: benzoyl peroxide, methyl methacrylate.	Powder: LOT J1352 Liquid: LOT I1608
ACRYPOL FAST	Ruthinium-Dental Manufacturing S.p.A., Rovigo, Italy	Fast heat curing acrylic resin with high molecular weight (PMMA) Liquid: methyl methacrylate, ethylene dimethacrylate. Powder: benzoyl peroxide, methyl methacrylate.	Powder: LOT I1160 Liquid: LOT H0759
IVOTION (control group)	Ivoclar vivadent, Schaan, Liechtenstein	PMMA Polymethyl methacrylate, pigments.	IBPink-YB5WNZ-117 IBPink-YB5WNZ-118
AADVA DISC (control group)	GC Corporation, Tokyo, Japan	PMMA.	NA
NEXTDENT DENTURE 3D+	NextDent B.V., Soesterberg, The Netherlands	3D-printed resin (PMMA) 2-hydroxyethyl methacrylate; diphenyl(2,4,6-trimethylbenzoyl) phosphine oxide; 2-hydroxyethyl methacrylate.	WW465N01
GC TEMP PRINT	GC Corporation, Tokyo, Japan	Urethane dimethacrylate (UDMA) dimethacrylate component ** quartz (SiO_2_) photoinitiator synergist UV-light absorber.	2,212,091
GC TEMP PRINT+ Pink	GC Corporation, Tokyo, Japan	Urethane dimethacrylate (UDMA) dimethacrylate component ** quartz (SiO_2_) photoinitiator synergist UV-light absorber Urethane dimethacrylate methacrylate monomer(s) photoinitiator(s).	NA
SprintRayEU Denture Base	SprintRay Inc., Los Angeles, CA, USA	3D-printed resin Ethoxylated bisphenol A dimethacrylate (BisEMA) 7,7,9 (or 7,9,9)-trimethyl-4,13-dioxo-3,14-dioxa-5,12- diazahexadecane-1,16-diyl bismethacrylate, 2-hydroxyethyl methacrylate, Silicon dioxide, diphenyl(2,4,6-trimethylbenzoyl)phosphine oxide, Titanium dioxide.	50,920,226

The substance is marked with **, then substance is a trade secret.

**Table 2:** Analog resins with corresponding polymerization technique used.

Name	Polymerization
ACRYPOL R ACRYPOL LL	Place the flask in water at room temperature until completely immersed. Heat the water in about 40/45 min at 70 °C, keep this temperature for 30 min, then bring the water to a boil and keep it for 30 min, then let it cool slowly in the water for another 30 min. Then, removing it from the water, allow the muffle to cool to room temperature.
ACRYPOL HI	Place the flask in water at room temperature until completely immersed. Slowly bring the water to the boil in at least 45 min. Simmer for 30 min, then leave to cool slowly in the water for another 30 min. Then, removing it from the water, cool the muffle at room temperature.
ACRYPOL FAST	Heat cured in boiling water for 20 min, remove the flask from water and leave it to cool at room temperature. Then, restart the device and bring the water to the boil and keep the temperature constant for 20 min. Remove from the water and cool to room temperature.
ACRYSELF ACRYSELF P	Polymerization at a temperature of 22 °C/23 °C starts approximately 12–15 min after mixing. It is recommended to carry out polymerization in a pot at a pressure of 2 ATM (standard atmosphere) for 10 min at a temperature of 45 °C.

**Table 3:** 3D-printed resins with corresponding polymerization technique used.

Name	Polymerization
NEXTDENT DENTURE 3D+	Polymerization with “LaboLight DUO” curing unit for 20 min.
NEXTDENT DENTURE 3D+ BB 40”	Polymerization with BB cure unit for 40 min.
SPRINTRAYEU DENTURE BASE	Polymerization with “LaboLight DUO” curing unit for 20 min.
SPRINTRAYEU DENTURE BASE BB 40”	Polymerization with BB cure unit for 40 min.
GC TEMP PRINT	Polymerization with “LaboLight DUO” curing unit for 20 min.
GC TEMP PRINT BB 20”	Polymerization with BB cure unit for 20 min.
GC TEMP PRINT BB 40”	Polymerization with BB cure unit for 40 min.
GC TEMP PRINT PINK BB 20”	Polymerization with BB cure unit for 20 min.
GC TEMP PRINT PINK BB 40”	Polymerization with BB cure unit for 40 min.

**Table 4:** Mean flexural strengths (mean), standard deviations (SD) and significative differences (Sign).

Resin Type	Mean (MPa)	SD	Sign
ACRYPOL R	89.15	14.31	adghij
ACRYSELF P	86.07	7.09	adghi
ACRYSELF	74.83	7.84	di
ACRYPOL HI	85.58	8.60	adghie
ACRYPOL LL	92.39	17.18	ghj
ACRYPOL FAST	98.86	10.66	hcj
IVOTION	91.88	4.43	egcj
AADVA DISC	107.87	7.56	cj
NEXTDENT LABO LIGHT 20”	60.11	5.72	b
SPRINTRAY LABO LIGHT 20”	54.07	3.55	b
TEMP PRINT LABO LIGHT 20”	75.58	9.36	i
TEMP PRINT PINK BB 20”	95.39	9.49	cghj
TEMP PRINT PINK BB 40”	102.96	9.37	j
TEMP PRINT BB 20”	90.87	7.44	aghj
NEXTDENT BB 40”	83.32	8.38	adgi
TEMP PRINT BB 40”	96.87	6.27	aghcj
SPRINTRAY BB 40”	85.44	5.30	adghij
ANOVA value *p*	F = 24.421 0.000		

Same letters per table denote no statistically significant differences (*p* > 0.05).

## Data Availability

The data presented in this study are available on request from the corresponding author. The data are not publicly available due to University policy.
